# Characterization of Long Non-Coding RNAs in Systemic Sclerosis Monocytes: A Potential Role for PSMB8-AS1 in Altered Cytokine Secretion

**DOI:** 10.3390/ijms22094365

**Published:** 2021-04-22

**Authors:** Nila H. Servaas, Barbara Mariotti, Maarten van der Kroef, Catharina G. K. Wichers, Aridaman Pandit, Flavia Bazzoni, Timothy R. D. J. Radstake, Marzia Rossato

**Affiliations:** 1 Center for Translational Immunology, University Medical Center Utrecht, Utrecht University, 3584 CX Utrecht, The Netherlands; nila.servaas@gmail.com (N.H.S.); maartenkroef1988@gmail.com (M.v.d.K.); R.Wichers@umcutrecht.nl (C.G.K.W.); A.Pandit@umcutrecht.nl (A.P.); tradstake73@gmail.com (T.R.D.J.R.); 2 University Medical Center Utrecht, Department of Rheumatology and Clinical Immunology, Utrecht University, 3584 CX Utrecht, The Netherlands; 3 Division of General Pathology, Department of Medicine, University of Verona, 37134 Verona, Italy; barbara.mariotti@univr.it (B.M.); flavia.bazzoni@univr.it (F.B.); 4 Department of Biotechnology, University of Verona, 37134 Verona, Italy

**Keywords:** systemic sclerosis, monocytes, long non-coding RNA, autoimmune, co-expression network

## Abstract

Systemic sclerosis (SSc) is a chronic autoimmune disease mainly affecting the connective tissue. In SSc patients, monocytes are increased in circulation, infiltrate affected tissues, and show a pro-inflammatory activation status, including the so-called interferon (IFN) signature. We previously demonstrated that the dysregulation of the IFN response in SSc monocytes is sustained by altered epigenetic factors as well as by upregulation of the long non-coding RNA (lncRNA) NRIR. Considering the enormously diverse molecular functions of lncRNAs in immune regulation, the present study investigated the genome-wide profile of lncRNAs in SSc monocytes, with the aim to further unravel their possible role in monocyte dysregulation and disease pathogenesis. Transcriptomic data from two independent cohorts of SSc patients identified 886 lncRNAs with an altered expression in SSc monocytes. Differentially expressed lncRNAs were correlated with neighboring protein coding genes implicated in the regulation of IFN responses and apoptotic signaling in SSc monocytes. In parallel, gene co-expression network analysis identified the lncRNA PSMB8-AS1 as a top-ranking hub gene in co-expression modules implicated in cell activation and response to viral and external stimuli. Functional characterization of PSMB8-AS1 in monocytes demonstrated that this lncRNA is involved in the secretion of IL-6 and TNFα, two pivotal pro-inflammatory cytokines altered in the circulation of SSc patients and associated with fibrosis and disease severity. Collectively, our data showed that lncRNAs are linked to monocyte dysregulation in SSc, and highlight their potential contribution to disease pathogenesis.

## 1. Introduction

Systemic sclerosis (SSc) is a chronic autoimmune disease with a highly heterogeneous clinical phenotype [1]. The disease is characterized by three main hallmarks: vascular abnormalities, immune system dysregulation, and fibrosis. Based on the extent of skin fibrosis and the presence of vascular and immunological abnormalities, SSc patients can be divided into four subsets: early SSc (eaSSc), non-cutaneous SSc (ncSSc), limited-cutaneous SSc (lcSSc), and diffuse-cutaneous SSc (dcSSc) [2,3]. Vascular abnormalities characterize the pre-clinical stage of SSc, and Raynaud’s Phenomenon (RP) occurs in 90–98% of patients with SSc, often preceding the disease onset by years [4]. However, the exact immunopathogenic mechanisms leading to the onset and contributing to the progression of SSc remain to be elucidated. Vascular injury and endothelial cell activation appear to be the earliest events in SSc pathogenesis [5]. This vascular damage is hypothesized to lead to the recruitment and activation of various immune cell types including lymphocytes, dendritic cells, and monocytes, which secrete various pro-inflammatory cytokines and growth factors such as IL-6, IL-8, IL-13, TNFα, TGFβ, and MCP-1 [6]. The resulting mix of inflammatory mediators induces the differentiation of resident epithelium, endothelium, and fibroblasts into myofibroblasts that deposit excessive amounts of extracellular matrix, leading to fibrosis and permanent tissue scarring [6].

Several lines of evidence implicate monocytes as an important cell type in SSc pathogenesis. Monocytes are among the predominant infiltrating mononuclear cells in SSc skin lesions [7,8,9], suggesting that these cells are involved in the fibrotic processes underlying the disease. In addition, the population of circulating monocytes is increased in the peripheral blood of SSc patients, and their frequency is correlated with the extent of skin fibrosis and the occurrence of interstitial lung disease (ILD) [10,11]. Besides their increased frequencies, SSc monocytes also display signs of enhanced activation, evident from an increased expression of interferon (IFN) responsive genes (referred to as the type I IFN signature) [12,13], and an enhanced production of pro-inflammatory and pro-fibrotic mediators [14,15,16]. Together, this evidence suggests a critical role for monocytes in the pathogenesis of SSc, linking immune aberrances and fibrosis.

Epigenetic [17,18] and miRNA-associated [13] alterations have been proposed as potential contributors to monocyte dysregulation in SSc. Next to these, long non-coding RNAs (lncRNAs) have recently gained widespread attention as critical biological regulators of gene expression in immune cells including monocytes [19] and have been linked to SSc pathogenesis [20,21,22,23,24]. lncRNAs are broadly defined as RNA transcripts longer than 200 nucleotides that lack protein coding capacity. They are involved in virtually all levels of gene expression regulation through a variety of biological mechanisms [25]. Based on the genomic localization relative to their targets, lncRNAs can be categorized as *cis*- or *trans*-acting, regulating the expression of neighboring or distal protein coding genes [26]. Additionally, the subcellular localization of lncRNAs also underlies their function [27]. Chromatin-associated and nuclear lncRNAs are often involved in the regulation of transcriptional processes, for example, through chromatin remodeling or the recruitment of transcription factors [28], while cytoplasmic lncRNAs most frequently act on post-transcriptional levels, for example, through miRNA sponging, the regulation of mRNA translation, or the alteration of protein activity [29]. 

We recently demonstrated that the lncRNA NRIR plays an important role in the regulation of type I IFN responses in monocytes [20]. NRIR is upregulated in SSc monocytes and promotes IFN-related pathways, thereby contributing to the type I IFN signature observed in these cells [20]. Because of the broad molecular functions of lncRNAs and their involvement in immune system regulation, we hypothesized that more lncRNAs may be implicated in the altered molecular processes characterizing monocytes of SSc patients. Exploiting transcriptomic data of monocytes obtained from SSc patients and matched healthy controls, we identified multiple lncRNAs potentially involved in the regulation of apoptotic pathways and IFN signaling in SSc monocytes. In addition, combining in silico and in vitro approaches, we identified the lncRNA PSMB8-AS1 as a potential regulator of cytokine release in SSc monocytes.

## 2. Results

### 2.1. The Expression of lncRNAs Is Altered in SSc Monocytes and Is Correlated with Neighboring Protein Coding Genes

The genome-wide expression profile of lncRNAs in healthy and SSc monocytes was initially assessed in the “Definite SSc cohort”, comprising ncSSc (n = 7), lcSSc (n = 11) and dcSSc (n = 7) patients, and matched healthy controls (n = 9, Table 1). A total of 886 lncRNAs were found to be differentially expressed in at least one group of SSc patients versus healthy controls (log_2_(FC) >0.58 or <−0.58, and *p*-value < 0.05) (Figure S1A, Table S1). Of these, 22 lncRNAs were commonly altered in all SSc subsets (Figure S1B). 

**Figure 1:**
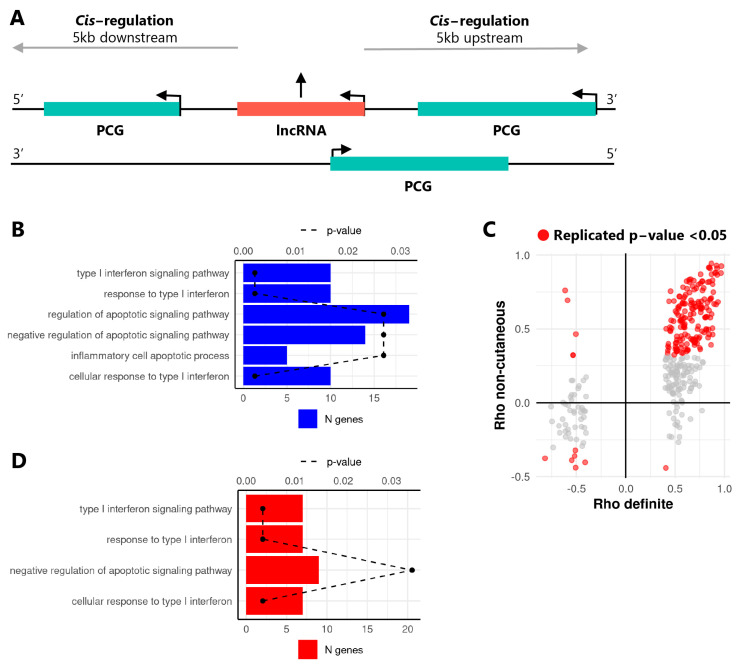
Differentially expressed lncRNAs are correlated with *cis* localized protein coding genes relevant for SSc pathogenesis. (**A**) Schematic overview of the *cis* correlation approach to identify neighboring long non-coding RNAs (lncRNA, red) and protein coding genes (PCGs, green). Arrows indicate the direction of transcription. (**B**) GO-term enrichment analysis results of PCGs identified in the *cis* correlation analysis in the Definite SSc cohort. GO terms for significantly enriched biological processes are given on the *y*-axis. Bars depict the number of genes identified within the enriched pathway (N genes, bottom *x*-axis), dashed line indicates B&H corrected *p*-value of the enrichment (*p*-value, top *x*-axis). (**C**) Correlation coefficients (Spearman’s Rho) between lncRNAs and PCGs in the Definite (*x*-axis) and Non-cutaneous (*y*-axis) cohorts. Each dot represents a lncRNA-PCG pair. Grey dots represent pairs significantly correlated in the Definite cohort only, while red dots represent pairs significantly correlated in both cohorts (Spearman’s rho >0.4 or <−0.4, and *p*-value ≤ 0.05). (**D**) GO-term enrichment analysis results of protein coding genes from the in *cis* correlation analysis replicated the Non-cutaneous cohort.

Since lncRNAs often regulate the transcription of neighboring protein coding genes (PCGs) [30], in *cis* correlation analysis was performed to identify putative target genes of differentially expressed lncRNAs in the Definite cohort. To this end, the expression levels of differentially expressed lncRNAs were correlated with PCGs located 5 kb upstream or downstream of each lncRNA gene (Figure 1A). Out of 886 differentially expressed, 278 lncRNAs were significantly correlated with PCGs localized in *cis* (Spearman’s rho >0.4 or <−0.4, and *p*-value ≤ 0.05), allowing for the identification of 332 lncRNA-PCG pairs. Functional enrichment analysis of the correlated PCGs identified pathways associated with IFN response, negative regulation of apoptosis, and inflammatory cell apoptotic processes (Figure 1B), indicating that lncRNAs altered in SSc monocytes potentially regulate genes involved in these pathways.

In order to substantiate these results and identify putative lncRNA-PCG pairs involved already at the early stages of SSc development, we repeated the *cis* correlation analysis in an additional cohort comprising ncSSc patients and SSc patients at the early disease stage (eaSSc), as well as individuals with Raynaud’s Phenomenon (RP) (“Non-cutaneous cohort”, Table 1). This analysis highlighted that 143 out of 332 correlated lncRNA-PCG pairs identified in the Definite cohort were reproduced in the Non-cutaneous cohort (Spearman’s rho >0.4 or <−0.4, and *p*-value ≤ 0.05, Figure 1C). GO-term enrichment analysis of the PCGs identified in the replicated pairs showed a significant enrichment for GO terms related to type I IFN and apoptosis (Figure 1D), suggesting that lncRNAs altered during SSc development are implicated in these processes. The 15 lncRNA-PCG pairs that were annotated in these biological pathways are given in Table 2.

### 2.2. Weighted Gene Co-Expression Network Analysis Identifies Clusters of Tightly Correlated RNAs Associated to SSc Clinical Features and Relevant Biological Processes

**Figure 2:**
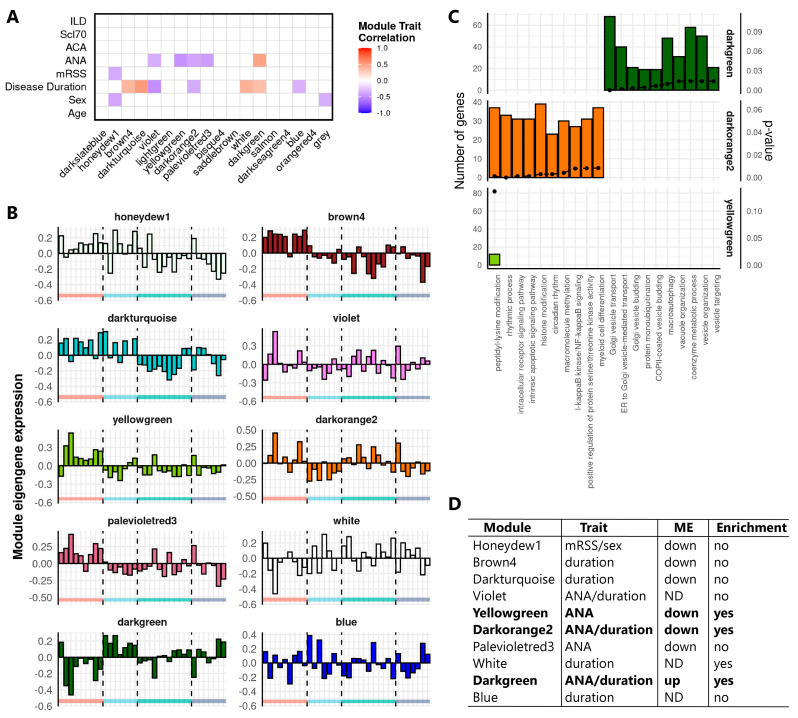
Identification and functional annotation of co-expression modules correlated with clinical traits in SSc. (**A**) Correlation of module eigengenes (MEs) to SSc clinical traits. Rows indicate the clinical traits, and columns indicate modules identified in the Definite cohort. Cells of significant correlations (Pearson, *p*-value < 0.05) are color-coded by the degree and direction of the correlation (red = positive; blue = negative). Abbreviations: mRSS, modified Rodnan Skin Score; ANA, antinuclear antibodies; ACA, anticentromere antibodies; Scl70, anti-topoisomerase I antibodies; ILD, interstitial lung disease. (**B**) ME expression (first principal component, *y*-axis) of modules significantly correlated with clinical traits. Bars represent individual donors grouped according to their disease subset represented by the colors on the *x*-axis (red = HC, light blue = ncSSc, green = lcSSc, dark blue = dcSSc). (**C**) GO-term enrichment analysis of selected modules. Top 10 enriched terms for each module are shown (B&H corrected *p*-value < 0.05). Bars depict the number of module genes associated to enriched GO terms, and dots represent the *p*-value for the enrichment. (**D**) Table indicating the characteristics for selected modules (Column 1), considering correlations to clinical traits (Column 2), distinct ME expression pattern versus heathy controls (Column 3, up = higher ME in SSc, down = lower ME in SSc, and ND = ME not distinct from healthy subjects), and functional enrichment (Column 4). Modules that were selected for subsequent analysis are highlighted in bold.

Next to *cis* regulatory lncRNAs, *trans*-acting lncRNAs are also emerging as important regulators of gene expression, especially at the post-transcriptional level [31]. To explore the regulatory potential of *trans*-acting lncRNAs in SSc monocytes, genome-wide co-expression network analysis was performed in parallel to the *cis* correlation analysis. Weighted gene co-expression network analysis (WGCNA) generated 18 distinct co-expression modules of highly correlated genes in the Definite cohort (Table S2). Correlation between module eigengenes (MEs) and clinical traits for SSc identified 10 co-expression modules that were significantly correlated with clinical parameters associated with SSc (Figure 2A, Pearson correlation, *p*-value < 0.05). Comparison of the global ME expression across SSc patients and healthy controls showed that the honeydew1, brown4, darkturquoise, yellowgreen, darkorange2, and paleviolet modules were lower in SSc patients, while the ME expression of the darkgreen module was higher in SSc patients (Figure 2B). Since the remaining violet, white, and blue modules did not show a distinct ME expression pattern in SSc patients, they were not considered for subsequent analysis (Figure 2B). Next, GO-term enrichment analysis was performed to annotate the seven modules with a distinct ME expression pattern in SSc patients. The darkgreen module was linked to vesicle transport and the regulation of autophagy, while the darkorange and yellowgreen modules were linked to IkappaB-NF-kappaB signaling, myeloid cell differentiation, and protein modifications (Figure 2C). No significant enrichment was identified for the remaining modules that were therefore not considered for further investigation (Figure 2D).

### 2.3. Identification of the lncRNA PSMB8-AS1 as a Reproducible Hub Gene Relevant for SSc and Monocyte Biology

**Figure 3:**
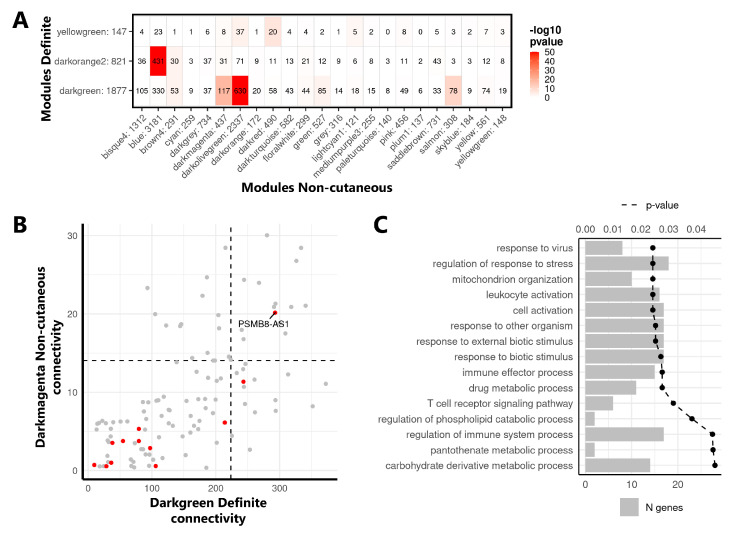
Identification of PSMB8-AS1 as a hub gene in replicated network modules. (**A**) Overlap of selected modules from the Definite (rows) and Non-cutaneous cohorts (columns). Numbers behind module names indicate the total number of genes in the modules. Numbers in the table indicate the number of genes overlapping between two modules. Coloring indicates the significance of the overlap (Fisher’s exact test, −10log (*p*-value)). (**B**) Intramodular connectivity of genes shared across the darkgreen module of the Definite cohort (*x*-axis) and the darkmagenta module of the Non-cutaneous cohort (*y*-axis). Each dot represents one gene, with lncRNAs highlighted in red. The top 25% most connected genes in both cohort modules (threshold indicated by black dashed lines) were considered as hub genes. (**C**) The GO-term enrichment of genes replicated across the darkgreen/darkmagenta modules. Bars depict the number of genes identified in the enrichment, the dotted line represents the B&H corrected *p*-value. Top 15 enriched terms are shown (B&H corrected *p*-value < 0.05).

The co-expression network analysis was repeated in the Non-cutaneous cohort, and the extent of overlap between the modules from the Definite and Non-cutaneous cohorts was assessed to identify reproducible co-expression modules (Figure S2, Table S3). Thirteen modules from the Non-cutaneous cohort showed a significant overlap with the 3 selected modules from the Definite cohort (i.e., darkgreen, darkorange, and yellowgreen, Fisher’s exact test, *p*-value < 0.05, Figure 3A), demonstrating that clinically relevant modules are reproducible across different cohorts of SSc monocytes, including pre-clinical SSc stages.

Next, by comparing the intramodular connectivity of shared genes across the overlapping modules (Figure S3), we identified replicated hub genes with high connectivity within modules across both cohorts. While several lncRNAs were present in the replicated modules, PSMB8-AS1 was the only lncRNA identified among the top 25% most highly connected genes (Figure 3B). Specifically, PSMB8-AS1 was a replicated hub gene in the darkgreen and darkmagenta modules from the Definite and Non-cutaneous networks, respectively, of which overlapping genes are enriched in genes related to immune cell activation and to response to virus and external stimulus (Figure 3C). Overall, these results pointed to PSMB8-AS1 as a potential central player in the regulation of these molecular pathways that are also relevant processes for monocyte activation in SSc.

### 2.4. Characterization of PSMB8-AS1 Expression in SSc and Healthy Monocytes

**Figure 4:**
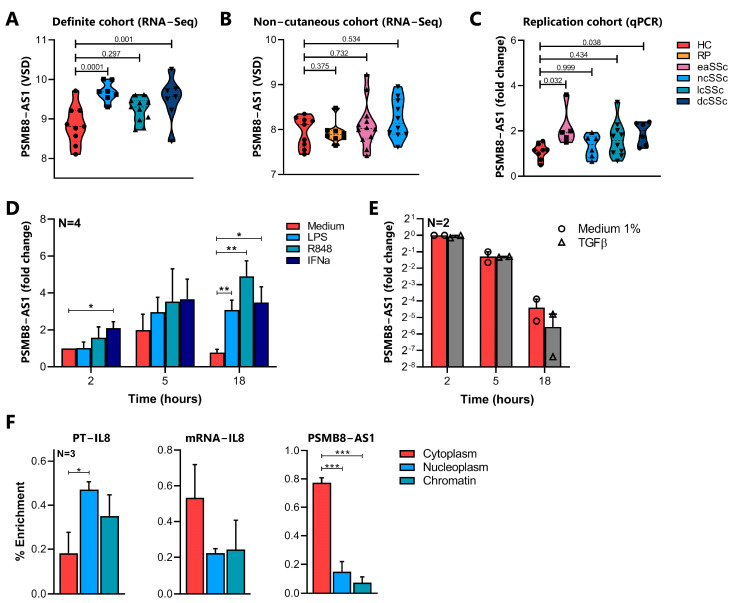
Molecular characterization of PSMB8-AS1 in healthy and SSc monocytes. PSMB8-AS1 expression in the (**A**) Definite cohort and (**B**) Non-cutaneous cohort assessed by RNA-Seq. Data are expressed as variance stabilized data (VSD), mean +/− SEM is reported. For each comparison the *p*-value, calculated according to the Wald test, is shown. (**C**) PSMB8-AS1 expression was analyzed in the “Replication cohort” by RT-qPCR. Data are reported as the fold change of each donor versus one representative healthy control, and mean +/− SEM is reported. *p*-values, as determined by the Kruskall–Wallis test with post-hoc Dunn’s test, are reported. (**D**) CD14+ monocytes were cultured for the indicated time points in the presence of LPS (100 ng/mL, light blue bars), R848 (5 µM, green bars), IFNα-a2 (1000 U/mL, dark blue bars), or (**E**) TGFβ (0.01 ng/µL, grey bars), or left untreated (medium control, red bars). PSMB8-AS1 expression was analyzed by RT-qPCR and expressed as a fold change over the medium control at 2 h. Data are shown as mean +/− SEM of (**D**) 4 and (**E**) 2 experiments. For (**D**), significance is indicated as * *p <* 0.05, ** *p <* 0.01 according to two-way ANOVA followed by Dunnett’s post-hoc test. (**F**) RNA from cytoplasm (red), nucleoplasm (blue), and chromatin (green) of CD14+ monocytes were obtained as described in Materials & Methods. Expression of IL-8 primary transcript (PT-IL8), IL-8 mRNA, and PSMB8-AS1 in each fraction was analyzed by RT-qPCR. Data are reported as a percentage of transcript in each compartment compared to total cell lysates (2^−∆CT*100, indicated by % Enrichment). Mean +/− SEM of 3 different experiments is shown. * *p <* 0.05, ***p < 0.001 according to one-way ANOVA followed by Tukey’s post-hoc test.

Both the *cis* correlation and co-expression network analysis highlighted PSMB8-AS1 as a putative key regulator of biological processes relevant for SSc pathogenesis and monocyte activation. PSMB8-AS1 expression was significantly upregulated in ncSSc and dcSSc patients in the Definite cohort (Figure 4A). A trend for PSMB8-AS1 upregulation was also observed in the lcSSc patients in the Definite cohort and in eaSSc and ncSSc patients of the Non-cutaneous cohort (Figure 4A,B). To confirm the altered expression of PSMB8-AS1 across SSc patients, its expression was evaluated by a target specific RT-qPCR in an additional, independent cohort of SSc patients (Replication cohort). A statistically significant altered expression of PSMB8-AS1 was confirmed in SSc patients with the earliest symptoms (eaSSc) and the most severe phenotype (dcSSc patients) (*p*-value < 0.05, Figure 4C). In the other SSc groups, only some individuals displayed the upregulation of this lncRNA, but this, however, was not significantly changed when considering the whole group.

To identify the molecular pathways leading to PSMB8-AS1 induction in SSc, its expression was assessed in healthy human monocytes cultured for 2, 5, and 18 h in the presence of LPS (TLR4 ligand), R848 (TLR7/8 ligand), IFNα, and TGFβ, all stimuli linked to monocyte activation and fibrosis in SSc [32,33,34,35]. PSMB8-AS1 expression was strongly induced by LPS, R848, and IFNα, especially after 18 h of stimulation (Figure 4D, *p*-value < 0.05 compared to untreated cells). In contrast, PSMB8-AS1 expression was not altered by TGFβ treatment (Figure 4E; control for TGFβ stimulation is given in Figure S4). All together, these results demonstrated that PSMB8-AS1 expression can be induced by pro-inflammatory stimuli relevant for SSc and possibly indicate its implication in the regulation of the downstream pathways, as previously demonstrated for other lncRNAs induced by pro-inflammatory stimuli [20].

Next, as the intracellular localization of lncRNAs partially governs their function [36], the specific cellular location of PSMB8-AS1 was determined by subcellular fractionation of healthy monocytes (Figure 4F). The percentage of enrichment of IL-8 Primary Transcript and IL-8 mRNA were used as positive controls to verify the appropriate separation of different compartments, as they are expected to be found in the nucleus/chromatin or cytoplasm, respectively. In agreement with previous findings in epithelial, glioma, and pancreatic cells [37,38,39], PSMB8-AS1 was found to be enriched in monocyte cytoplasm (Figure 4F, third panel), suggesting that this lncRNA may regulate expression of its target(s) at the post-transcriptional level [29].

### 2.5. Characterization of PSMB8-AS1 Function in Monocytes

**Figure 5:**
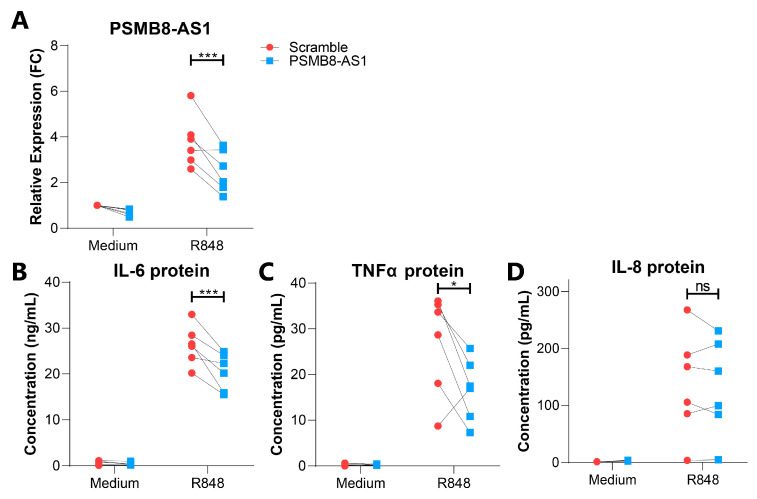
PSMB8-AS1 is involved in the regulation of cytokine levels in CD14+ monocytes. (**A**) CD14+ monocytes were transfected with si-PSMB8-AS1 (blue) or scramble siRNA (red), and stimulated with R848 for 18 h or left untreated (medium control). The expression of PSMB8-AS1 was analyzed by RT-qPCR and expressed as relative expression (FC = fold change) compared to medium scramble control. Protein levels of IL-6 (**B**), TNFα (**C**), and IL-8 (**D**) in the cell free supernatants of transfected monocytes were determined by ELISA. * = *p*-value < 0.05, *** = *p*-value < 0.001, ns = not significant, as determined by two-way ANOVA followed by Bonferroni’s post-hoc test.

To further investigate the biological relevance of PSMB8-AS1, its expression was efficiently silenced in resting and R848 stimulated monocytes using a specific small interfering RNA (siRNA) (Figure 5A, *p* < 0.001). The impact of PSMB8-AS1 silencing on monocyte apoptosis was first investigated, as this lncRNA has previously been reported as a negative regulator of apoptotic signaling [37,38,39], and apoptotic signaling pathways were also identified by our GO-term enrichment analysis in the *cis* correlation analysis. However, FACS analysis demonstrated that PSMB8-AS1 silencing in monocytes did not affect apoptosis, neither in resting nor stimulated conditions (*p*-value > 0.05, Figure S5A,B). Moreover, PSMB8-AS1 silencing also did not affect the expression of its *cis* correlated PCG PSMB8 (Figure S5C), showing that this lncRNA does not function via *cis* regulatory mechanisms, at least in the conditions tested here. 

As the co-expression network analysis identified PSMB8-AS1 as a potential *trans*-acting hub lncRNA implicated in the regulation of immune cell activation and vesicle-related transport, its role in the secretion of pro-inflammatory cytokines was subsequently investigated. To this end, the impact of PSMB8-AS1 silencing was assessed on IL-6, IL-8, and TNFα secretion, three cytokines released by activated monocytes, and at higher levels by stimulated SSc monocytes [15,16]. ELISA analysis showed a significant decrease of IL-6 and TNFα protein concentrations in the cell-free supernatant of si-PSMB8-AS1 monocytes stimulated with R848 for 18 h as compared to monocytes transfected with scramble siRNA (Figure 5B,C), whereas IL-8 protein levels were not affected (Figure 5D). Overall, these results demonstrated that PSMB8-AS1 regulates the secretion of specific cytokines in TLR-activated monocytes, and can thereby contribute to monocyte activation in SSc.

## 3. Discussion

lncRNAs have been described as pivotal biological regulators of immune responses [19], and previous studies have suggested a role for lncRNAs in SSc [20,21,22,23,24]. However, a thorough characterization of the lncRNA profile in specific cell subsets obtained from SSc patients is still lacking. The aim of this study was to investigate the potential role of lncRNAs in the dysregulation of SSc monocytes, an important cell type implicated in SSc pathogenesis. *Cis* correlation and co-expression network analysis demonstrated that numerous lncRNAs are altered in SSc monocytes and are potentially implicated in the regulation of various biological processes relevant for SSc pathogenesis. 

We have previously shown that the lncRNA NRIR plays an important role in IFN responses of SSc monocytes [20]. The results reported in the present study reinforce the concept that, besides NRIR, other lncRNAs might be strongly implicated in the regulation of IFN responses in these cells. Indeed, we identified a correlation between RP11-326I11.3, a lncRNA highly expressed in both the Definite and Non-cutaneous cohort, and IRF2, an important regulator of IFN responses [40]. Interestingly, a correlation between these two genes has previously been described in brain tissue, where they were linked to IFN signaling in central nervous system homeostasis [41]. Currently available clinical and molecular data suggest that type I IFN dysregulation is a major contributor to SSc pathogenesis [34], and the augmented expression of Type I IFN inducible genes in SSc monocytes has been linked to inflammation and the development of fibrosis [12]. Since our results showed that multiple lncRNAs are potentially involved in the amplified IFN signaling in SSc monocytes, further investigations should address the possible implication of these molecules in the disease pathogenesis. 

Next to IFN signaling, *cis* correlated lncRNA-PCG pairs also linked this class of regulators to the negative regulation of apoptosis, a relevant process that, if inhibited, can potentially contribute to the increased numbers of circulating monocytes observed in SSc patients. RP11-356I2.4, for example, was strongly correlated with TNFAIP3, an apoptosis regulator that is induced through NF-κB signaling [42]. A correlation between RP11-356I2.4 and TNFAIP3 has also previously been observed in the inflammatory skin disorder chronic actinic dermatitis, where both genes were downregulated in comparison to healthy controls [43]. As actinic dermatitis, like SSc, is also characterized by inflamed and thickened skin, lncRNAs involved in monocyte apoptotic processes could potentially contribute to fibrotic processes in the skin. 

Both the *cis* correlation analysis and co-expression network analysis pointed at the lncRNA PSMB8-AS1 as a key regulator of altered gene-expression in SSc monocytes. Although this lncRNA has previously been described in the context of influenza infection [44] and cancer [37,38,39], our results link PSMB8-AS1 dysregulation with autoimmunity for the first time. The *cis* correlation analysis predicted PSMB8, the protein coding gene located antisense to PSMB8-AS1, as a potential target of this lncRNA; however, the two genes were not present in the same co-expression modules, and PSMB8 mRNA levels were not altered upon PSMB8-AS1 silencing. These two genes are therefore not functionally related within SSc monocytes, while their expression correlation is probably the result of a shared transcriptional program given that their promoters share a binding site for the IFN inducible transcription factor IRF1 [45]. Consistently, the protein PSMB8 can be directly regulated by IRF1 [46], and treatment of monocytes with IFNα and R848 (a TLR7/8 ligand activating IFN signaling) induces PSMB8-AS1 expression, an event likely mediated by IRF1. These findings demonstrated that IFN-mediated activation is, at least partially, responsible for the upregulation of PSMB8-AS1 in SSc monocytes, in agreement with previous studies showing that this lncRNA can be induced by influenza virus infections (triggering IFN signaling) in other human cells [44].

In line with previous observations of three other studies in pancreatic epithelial cell lines and glioma [37,38,39], our subcellular localization analysis demonstrated that PSMB8-AS1 is present in the cytoplasm of CD14+ monocytes. Given that cytoplasmic lncRNAs can regulate secretory and extracellular vesicles [47], and PSMB8-AS1 was present in co-expression modules annotated to vesicle related transport, it is possible that this lncRNA is involved in the regulation of cytoplasmic vesicles. A more detailed characterization by RNA-FISH of the precise subcellular compartments involved in intracellular vesicle transport (for example, the endoplasmic reticulum or Golgi) [36,48] could provide better insights into the exact molecular processes related to PSMB8-AS1 activity. In addition, since different stimuli can modify the subcellular compartmentalization of lncRNAs [49], it would be interesting to verify whether factors relevant for SSc pathogenesis (e.g., TLR-agonists, IFNα, and CXCL4) can influence PSMB8-AS1 localization. The evidence that IL-6 and TNFα protein secretion was repressed by PSMB8-AS1 silencing in monocytes demonstrated that this lncRNA is involved in the positive regulation of these cytokines and possibly links its cytoplasmic localization to the control of cytokine secretion. In contrast, IL-8 release was unaffected, suggesting a possible cytokine-specific action of PSMB8-AS1. This is not surprising, given that specific cytokines can be secreted through distinct pathways in macrophages, adapted to suit specific stimulatory conditions [50]. Alternatively, the different impact of PSMB8-AS1 silencing on IL-6/TNFα and IL-8 secretion could be explained by distinct timeframes in the synthesis/expression of these factors or could be dependent on the specific stimuli used in our experiments. More detailed molecular investigations, exploiting a wide range of pro-inflammatory mediators, as well as stimulation times, are required to unravel the exact role of PSMB8-AS1 in cytokine release.

The positive regulation of IL-6 and TNFα secretion by PMSB8-AS1 directly link this lncRNA to SSc pathogenesis. Elevated levels of IL-6 and TNFα have indeed been reported in the serum of SSc patients in several studies [51,52], and both cytokines are associated with fibrotic processes [53,54], disease progression, and the occurrence of interstitial lung disease [55,56], especially in dcSSc patients. Most importantly, different studies proposed monocytes as the potential source of these cytokines in SSc [15,16,57]. Interestingly, the highest upregulation of PSMB8-AS1 was observed in monocytes from dcSSc patients, where the increase of IL-6 and TNFα levels and the extent of skin fibrosis are the most severe. Even if the upregulation of PSMB8-AS1 was fluctuating in other disease subsets, possibly due to the extreme clinical heterogeneity of these patients, the upregulation of this lncRNA was also confirmed in early and ncSSc patients. Such evidence indicates that PSMB8-AS1 might be involved in the regulation of inflammatory processes from early stages of the disease onward, affecting the secretion of cytokines that eventually contribute to the development and perpetuation of fibrosis.

In conclusion, in-depth bioinformatics analysis unraveled numerous lncRNAs dysregulated in SSc monocytes and highlighted an important regulatory potential of these molecules both in immune activation and disease pathogenesis. Among these, we specifically discovered that the upregulation of PSMB8-AS1 can modulate the secretion of pro-inflammatory cytokines by monocytes, thereby potentially contributing to the increased activation of these cells in SSc. A more detailed understanding of lncRNAs and their contribution to disease pathogenesis could provide steppingstones for the identification of novel molecular targets for manipulating monocyte activity in SSc, in order to contrast disease onset and progression.

## 4. Materials and Methods

### 4.1. Patient Demographics

Transcriptomic data from SSc patients and matched healthy controls from the Definite and Non-cutaneous cohorts were obtained from the gene expression omnibus (GSE124075). For the replication cohort, peripheral blood samples from SSc patients and age/sex matched healthy controls were obtained from the University Medical Center Utrecht. All participants enrolled in the study signed an informed consent form approved by the local institutional review boards prior to inclusion in this study (METC no. 12-466C, approved 2 October 2012), adherent of the Declaration of Helsinki Principles. Samples and clinical information were treated anonymously immediately after collection. SSc patients fulfilled the ACR/EULAR classification criteria [2] and were classified according to the extent of their skin fibrosis as lcSSc or dcSSc patients. Patients that fulfilled the classification criteria but did not present skin fibrosis are referred to as ncSSc patients throughout the manuscript. Finally, we also included eaSSc patients with Raynaud’s Phenomenon and positivity for SSc-specific autoantibodies and/or typical nailfold capillaroscopy patterns, as defined by LeRoy et al. [3]. The demographics and clinical characteristics of the subjects enrolled in these cohorts are reported in Table 1. Ongoing treatment regimens are reported in Table S4.

### 4.2. Purification and Culture of CD14+ Monocytes from Healthy Control Blood and Buffy Coats

PBMCs were isolated from whole heparinized blood samples from SSc patients and healthy controls or from the buffy coats of healthy controls, by density gradient centrifugation using Ficoll-Paque Plus (GE Healthcare, Chicago, IL, USA). CD14+ monocytes were purified from PBMCs using the MACS Human Monocyte Isolation Kit II (Miltenyi Biotec, Bergisch Gladbach, Germany) on the autoMACs Pro Separator (Miltenyi Biotec) according to the manufacturer’s instructions. For subsequent analysis, only cell preparations with more than 95% purity (measured by FACS analysis) for CD14+ cells were used.

For selected experiments, CD14+ monocytes purified from buffy coats were cultured in RPMI 1640 + 10% FCS (fetal calf serum, <0.5 EU/mL, Sigma-Aldrich, St. Louis, MO, USA) + 2 mM Glutamine at a concentration of 2 × 10^6^ cells/mL. Cultured cells were left untreated (medium control) or treated with one of the following stimuli: 100 ng/mL ultra-pure *E. coli* lipopolysaccharide (LPS, strain O111:B4, Invivogen, San Diego, CA, USA), 5 µM R848 (Invivogen), 1000 U/mL IFNα-2a (Cell Sciences, Newburyport, MA, USA), and TGF-β2 (Bio-Techne, Minneapolis, MN, USA) according to the conditions and times indicated for each experiment in the results section.

### 4.3. RNA Purification

Total RNA was purified using the DNA/RNA/miRNA Universal kit (Qiagen, Hilden, Germany) according to the manufacturer’s instructions. DNAse treatment (RNAse Free DNase I set, Qiagen) on column was performed. RNA was quantified with the Qubit^®^ RNA Assay Kit (Life Technologies, Carlsbad, CA, USA) on the Qubit^®^ Fluorometer (Invitrogen, Carlsbad, CA, USA) or on the Nanodrop 2000 spectrophotometer (Thermo Scientific, Waltham, MA, USA).

### 4.4. RNA-Sequencing Analysis

Raw sequencing data was obtained from GSE124075 [17]. Sequencing reads were aligned to the GrCh38 reference human genome (Genome Reference consortium) and the *H. sapiens* transcriptome (Ensembl, version 77) using TopHat [58]. Summed exon read counts per gene were estimated using the HTSeq-count function provided in the HTSeq python package [59] (v. 0.6.1p1). Differential expression analysis was performed using the negative binomial distribution-based method implemented in DESeq2 [60] (v. 1.6.3), and pair wise comparisons between SSc patients and HC groups were tested using the Wald test. Genes with a log_2_(FC) ≥0.58 or ≤−0.58 and a *p*-value ≤ 0.05 were considered significantly modulated. Normalized gene expression levels were expressed as variance stabilized data (VSD), calculated according to DESeq2 instructions. Gene types were annotated according to the Ensembl 77 database.

### 4.5. In Cis Correlation Analysis

Protein-coding genes (PCGs) that were localized within a region of 5 kb upstream or 5 kb downstream, regardless of the sense of transcription, for each differentially expressed lncRNA were recovered using the Biomart tool available on the Ensembl website. A Spearman rank-order correlation analysis of the expression of the lncRNAs and associated PCGs was then performed. Correlations with Spearman’s Rho ≥0.4 or ≤−0.4, and *p*-value ≤ 0.05 were considered significant. Correlation analysis was performed using the rcorr function implemented in the Hmisc package in R [61].

### 4.6. GO-Term Enrichment Analysis

Gene ontology (GO) enrichment analysis was performed using ToppFun [62]. Enrichment of biological process (BP) associated GO terms was tested using the probability density function. *p*-value was adjusted according to Benjamini–Hochberg/FDR correction. BP terms significantly enriched (B&H corrected *p*-value ≤ 0.05) were considered.

### 4.7. Weighted Gene Co-Expression Network Analysis (WGCNA)

Weighted gene co-expression networks were constructed for the Definite and Non-cutaneous cohorts separately using the R package WGCNA [63]. The VSD data of all genes with at least 1 count in all samples were used as input. An unsigned network with a scale-free topology was constructed, using a soft threshold power β = 13 for the Definite cohort and a soft threshold power β = 4 for the Non-cutaneous network. Modules were identified using the cutreeDynamic function with a minimum module size of 30. Closely related modules were merged using the mergeCloseModules function (cutHeight = 0.25). Gene expression profiles across the modules were summarized into module eigengene (ME) values based on the first principal component of each module. Fisher’s exact test was used to calculate the extent of module overlap between the Definite and Non-cutaneous networks, as previously described [64]. Intramodular connectivity (i.e., the connectivity of a gene to genes nodes within the same module) was obtained using the intramodularConnectivity function from the WGCNA package.

### 4.8. Subcellular Fractionation

CD14+ monocytes were harvested and resuspended in cold RLN1 solution (50 mM Tris HCl pH 8.0; 140 mM NaCl; 1.5 mM MgCl_2_; 0.5% NP-40) supplemented with RNAse and protease inhibitors (1 U/µL RNAse Out, 5 µg/mL leupeptin, 5 µg/mL pepstatin, 20 µM PAO, 1 mM PMSF, 1 mM Na_3_VO_4_, 50 mM NaF, and 10 mM DTT) and incubated for 15 min on ice. After centrifugation at 4 °C for 2 min at 200× *g*, the supernatant was saved as a “cytoplasmic fraction”. The pellet was washed in cold RLN1 and resuspended in cold RLN2 solution (50 mM Tris HCl pH 8.0; 500 mM NaCl; 1.5 mM MgCl_2_; 0.5% NP-40) supplemented with RNAse and protease inhibitors (as described for RLN1) and incubated for 10 min on ice. After centrifugation at 4 °C for 2 min at 500× *g*, the supernatant was saved as a “nuclear fraction”. The remaining pellet was washed in RLN1 solution and saved as a “chromatin fraction”. All fractions were resuspended in RLT+ (Qiagen) plus β-mercaptoethanol (Sigma-Aldrich, St. Louis, MO, USA) and processed for RNA extraction.

### 4.9. Transfection of CD14+ Monocytes Using siRNA

Purified CD14+ monocytes were transfected by means of electroporation, using the Amaxa™ Nucleofector™ II system (Lonza Basel, Switzerland) in combination with the Amaxa^®^ Human Monocyte Nucleofector^®^ Kit (Lonza). A minimum amount of 5 × 10^6^, up to a maximum amount of 15 × 10^6^ CD14+ monocytes were used for transfection, according to the manufacturer’s protocol. Monocytes were transfected with 200 pmol of Silencer Select Pre-Designed siRNA (assay id n503525, PSMB8-AS1, Ambion, Austin, TX, USA) or 200 pmol of Silencer Negative control No.1 siRNA (Ambion). After transfection, the cells were plated in 50% RPMI 1640 + 10% FCS + 2 mM Glutamine, and 50% Iscove’s Modified Dulbecco’s Medium (IMDM, Lonza) + 10% FCS + 2 mM Glutamine, at a concentration of 3 × 10^6^ cells/mL overnight. The next day, the medium was changed to RPMI 1640 + 10% FCS + 2 mM Glutamine, and the cells were stimulated for the times indicated for each separate experiment.

### 4.10. Reverse Transcription Quantitative Real-Time PCR (RT-qPCR)

Purified RNA (200–1000 ng) was reverse transcribed using the SuperScript^®^ III Reverse Transcriptase kit (Invitrogen), according to the manufacturer’s instructions. Gene expression was quantified, in duplicate, by RT-qPCR using 9 ng cDNA with SYBR Select Master Mix (Applied Biosystems, Foster City, CA, USA), in the presence of 400 nM gene-specific primers (Table 3), on the ViiA™ 7 Real-Time PCR System (Applied Biosystems). Relative expression of each gene was determined according to the comparative CT (ΔΔCT) method using RPL32 as an endogenous control (where the ΔCT equals the CT of the mRNA of interest—the CT of RPL32) [65].

### 4.11. FACS Assessment of Monocyte Viability

Viability of CD14+ monocytes was studied by FACS analysis. At room temperature, 200,000 cells were stained for 15 min in an Annexin binding buffer (Invitrogen) using the following antibodies: CD14-PE (clone M5E2, cat. 561707, BD Biosciences, Franklin Lakes, NJ, USA), CD16-V500 (clone 3G8, cat. 561393, BD Biosciences), 7-AAD (cat. 559925, BD Biosciences), and Annexin V-APC (cat. 550474, BD Biosciences). Sample fluorescence was measured on the LSRFortessa (BD Biosciences). Data were analyzed using FlowJo (Version 10, Tree Star Inc., Ashland, OR, USA).

### 4.12. Assessment of Cytokine Levels Using ELISA

Concentrations of IL-6, TNFα, and IL-8 in cell-free supernatants from cultured CD14+ monocytes were measured by the sandwich enzyme linked immunosorbent assay (ELISA). IL-6 and IL-8 were quantified using the PeliKine compact human IL-6 and IL-8 ELISA kits (Sanquin Reagents, Amsterdam, The Netherlands), and TNFα was quantified using the Human TNF-α ELISA Set (Diaclone, Besançon, France), according to the manufacturer’s instructions.

### 4.13. Statistical Analysis

Data are expressed as mean ± SEM unless otherwise indicated. Unless indicated otherwise, analysis of differences was performed using the Mann Whitney test. For multiple group comparisons, the one- or two-way analysis of variance (ANOVA) was used. *p*-values < 0.05 were considered statistically significant. Figures were produced using the R package ggplot2 [66] or the GraphPad Prism software (v 8.3, www.graphpad.com, GraphPad Software, Inc., San Diego, CA, USA).

## Figures and Tables

**Table 1:** Demographics and clinical features of subjects enrolled in the study. Values reported indicate the number (n) of patients and the median for each parameter (Interquartile Range (IQR), if not otherwise indicated). ACA, anticentromere antibodies; ANA, antinuclear antibodies; dcSSc, diffuse cutaneous SSc; eaSSc, early SSc; HC, healthy controls; ILD, Interstitial Lung Disease; Immunosupp., immunosuppressive therapy; lcSSc, limited cutaneous SSc; mRSS, modified Rodnan Skin Score; ncSSc, non-cutaneous SSc; NVC, nailfold videocapillaroscopy; pos, positivity; RP, Raynaud’s Phenomenon; Scl70, anti-topoisomerase antibodies; Tel., telangiectasia; yr., years. * = 1 patient unknown, ** = 6 patients unknown, *** = 3 patients unknown.

Definite Cohort	HC (9)	-	-	ncSSc (7)	lcSSc (11)	dcSSc (7)
Non-Cutaneous Cohort	HC (9)	RP (9)	eaSSc (11)	ncSSc (10)	-	-
Replication Cohort	HC (8)	-	eaSSc (5)	ncSSc (6)	lcSSc (10)	dcSSc (6)
Age (yr.)	52 (30–64)	-	-	45 (26–63)	59 (45–70)	58 (34–72)
	38 (28–49)	47(22–70)	57 (40–77)	52 (25–70)	-	-
	57 (31–64)	-	47 (22–61)	41 (36–55)	58 (38–69)	56 (53–72)
Female/Male, n	5/4	-	-	6/1	8/3	3/4
	9/0	9/0	11/0	10/0	-	-
	7/1	-	4/1	4/2	8/2	4/2
ANA, n (% pos.)	-	-	-	6 (86%)	10 (91%)	7 (100%)
	-	3 (33%)	10 (91%)	10 (100%)	-	-
	-	-	4 (80%)	6 (100%)	8 * (80%)	6 (100%)
ACA, n (% pos.)	-	-	-	3 (43%)	6 * (55%)	1 (14%)
	-	0 (0%)	7 (64%)	8 (80%)	-	-
	-	-	1 (20%)	1 (17%)	4 * (40%)	1 (17%)
Scl70, n (% pos.)	-	-	-	2 (29%)	2 * (18%)	4 (57%)
	-	0 (0%)	2 (18%)	1 (10%)	-	-
	-	-	1 (20%)	2 (33%)	2 * (20%)	4 (67%)
ILD, n (% pos.)	-	-	-	1 (14%)	2 (18%)	5 (71%)
	-	0 (0%)	0 (0%)	0 (0%)	-	-
	-	-	1 (20%)	2 (33%)	3 * (30%)	3 (50%)
mRSS	-	-	-	0	6 (0–12)	14 * (5–36)
	-	0	0	0	-	-
	-	-	0	0	4 * (2–14)	13 (4–23)
Tel., n (%)	-	-	-	3 * (43%)	4 (36%)	4 (57%)
	-	0 (0%)	1 (9%)	4 (40%)	-	-
	-	-	1 * (20%)	3 (50%)	6 * (60%)	2 * (33%)
NVC early, n (%)	-	-	-	2 * (29%)	2 ** (18%)	1 ***(14%)
	-	0 (0%)	9 (82%)	5 (50%)	-	-
	-	-	3 * (60%)	4 (66%)	3 ** (30%)	3 (50%)
NVC late/active,	-	-	-	4 * (57%)	3 ** (27%)	2 ***(28%)
n (%)	-	0 (0%)	0 (0%)	5 (50%)	-	-
	-	-	1 * (20%)	2 (33%)	1 ** (10%)	3 (50%)
Steroids, n (%)	-	-	-	0 (0%)	0 (9%)	2 (28%)
	-	0 (0%)	0 (0%)	0 (0%)	-	-
	-	-	1 * (20%)	0 (0%)	1 * (10%)	1 (17%)
Immunosup., n (%)	-	-	-	0 (0%)	1 (9%)	3 (43%)
	-	0 (0%)	1 (9%)	0 (0%)	-	-
	-	-	1 (20%)	2 (33%)	1 * (10%)	4 (66%)

**Table 2:** Replicated *cis* correlating lncRNA-PCG pairs are annotated in biological processes relevant for SSc. PCG, protein coding gene; lncRNA, long non-coding RNA; BM, base mean expression level; R, Spearman’s rank correlation coefficient; *p*, *p*-value.

			Definite Cohort			Non-Cutaneous Cohort		
GO-Term	PCG	lncRNA	BM	R	*p*	BM	R	*p*
Type I interferon response (GO:0071357, GO:0060337, GO:0034340)	PSMB8	PSMB8-AS1	645.23	0.67	0.000	262.01	0.68	0.000
	OAS1	RP1-71H24.6	46.71	0.50	0.003	17.74	0.62	0.000
	IRF2	RP11-326I11.3	75.09	0.61	0.000	40.19	0.73	0.000
	CACTIN	CACTIN-AS1	6.68	0.60	0.000	3.25	0.62	0.000
	IFITM3	RP11-326C3.11	14.01	0.58	0.000	9.65	0.68	0.000
	IFI6	RP11-288L9.4	16.93	0.41	0.017	7.28	0.50	0.001
	MX1	AP001610.5	7.46	0.59	0.000	8.20	0.72	0.000
	PAM16	RP11-295D4.3	30.27	0.88	0.000	40.74	0.80	0.000
Negative regulation of apoptotic signaling pathway (GO:2001234)	FAS	RP11-399O19.9	12.26	0.56	0.001	15.18	0.56	0.000
	BIRC6	AL133243.2	50.33	0.45	0.008	87.28	0.51	0.001
	THBS1	CTD-2033D15.2	40.78	0.76	0.000	76.70	0.87	0.000
	SGMS1	RP11-521C22.2	33.86	0.60	0.000	54.93	0.53	0.001
	CCAR2	RP11-582J16.5	34.78	0.59	0.000	64.17	0.67	0.000
	AATF	CTC-268N12.3	0.58	−0.41	0.015	0.56	−0.40	0.011
	TNAIP3	RP11-356I2.4	66.03	0.84	0.000	96.56	0.50	0.001
	IFI6	RP11-288L9.4	16.93	0.41	0.017	7.28	0.50	0.001

**Table 3:** Gene specific primer pairs used for RT-qPCR.

Gene	Forward Primer	Reverse Primer
PSMB8-AS1	CTTCTCTGCTCTCCCGTTATG	GTGTGTTACCTCCTTTCCAAG
RPL32	AGGGTTCGTAGAAGATTCAAGG	GGAAACATTGTGAGCGATCTC
IL-8	GCTCTGTGTGAAGGTGCAGT	CCAGACAGAGCTCTCTTCCA
PT-IL-8	ATTGAGAGTGGACCACACTG	ACTACTGTAATCCTAACACCTG
PSMB8	GAGGCGTTGTCAATATGTACC	CCTGGGGGAAATGCTTGTTC
MMP2	AGCGAGTGGATGCCGCCTTTAA	CATTCCAGGCATCTGCGATGAG

## Data Availability

RNA-sequencing data was obtained from the gene expression omnibus (GSE124075). All other relevant raw data from the data presented in the manuscript or the supplementary figures and tables are available by the authors of the study upon request.

## Supplementary Materials

The following are available online at https://www.mdpi.com/article/10.3390/ijms22094365/s1. Figure S1: Expression of lncRNAs is altered in SSc monocytes. Figure S2: Module overlap between gene co-expression networks identified in the Definite and Non-Cutaneous cohorts. Figure S3: Connectivity overlap of selected reproduced co-expression modules. Figure S4: MMP2 expression is induced by TGFβ signaling in monocytes. Figure S5: Silencing of PSMB8-AS1 does not affect apoptosis or PSMB8 expression in healthy monocytes. Table S1: Differentially expressed lncRNA identified in SSc monocytes. Table S2: Co-expression network analysis in the Definite cohort. Table S3: Module overlap analysis between the Definite and Non-cutaneous cohorts. Table S4: Ongoing treatment regimens of patients included in the study.

## References

[1] Allanore Y., Simms R., Distler O., Trojanowska M., Pope J., Denton C.P., Varga J. (2015). Systemic sclerosis. Nat. Rev. Dis. Prim..

[2] Van den Hoogen F., Khanna D., Fransen J., Johnson S.R., Baron M., Tyndall A., Matucci-Cerinic M., Naden R.P., Medsgen T.A., Carreira P.E. (2013). 2013 classification criteria for systemic sclerosis: An American college of rheumatology/European league against rheumatism collaborative initiative. Ann. Rheum. Dis..

[3] Leroy E.C., Medsger T.A. (2001). Criteria for the classification of early systemic sclerosis. J. Rheumatol..

[4] Belch J.J.F. (1991). Raynaud’s phenomenon: Its relevance to scleroderma. Ann. Rheum. Dis..

[5] Kahaleh M.B. (2004). Vascular involvement in systemic sclerosis (SSc). Clin. Exp. Rheumatol..

[6] Epattanaik D., Ebrown M., Postlethwaite B.C., Postlethwaite A.E. (2015). Pathogenesis of Systemic Sclerosis. Front. Immunol..

[7] Ishikawa O., Ishikawa H. (1992). Macrophage infiltration in the skin of patients with systemic sclerosis. J. Rheumatol..

[8] Kräling B.M., Maul G.G., Jimenez S.A. (1995). Mononuciear cellular infiltrates in clinically involved skin from patients with systemic sclerosis of recent onset predominantly consist of monocytes/macrophages. Pathobiology.

[9] Higashi-Kuwata N., Jinnin M., Makino T., Fukushima S., Inoue Y., Muchemwa F.C., Yonemura Y., Komohara Y., Takeya M., Mitsuya H. (2010). Characterization of monocyte/macrophage subsets in the skin and peripheral blood derived from patients with systemic sclerosis. Arthritis Res. Ther..

[10] Van der Kroef M., Hoogen L.L.V.D., Mertens J.S., Blokland S.L., Haskett S., Devaprasad A., Carvalheiro T., Chouri E., Vazirpanah N., Cossu M. (2019). Cytometry by time of flight identifies distinct signatures in patients with systemic sclerosis, systemic lupus erythematosus and Sjögrens syndrome. Eur. J. Immunol..

[11] Scott M.K.D., Quinn K., Li Q., Carroll R., Warsinske H., Vallania F., Chen S., Carns M.A., Aren K., Sun J. (2019). Increased monocyte count as a cellular biomarker for poor outcomes in fibrotic diseases: A retrospective, multicenter cohort study. Lancet Respir Med..

[12] Brkic Z., van Bon L., Cossu M., van Helden-Meeuwsen C.G., Vonk M.C., Knaapen H., Berg W.V.D., Dalm V.A., van Daele P.L., Severino A. (2016). The interferon type I signature is present in systemic sclerosis before overt fibrosis and might contribute to its pathogenesis through high BAFF gene expression and high collagen synthesis. Ann. Rheum. Dis..

[13] Ciechomska M., Wojtas B., Swacha M., Olesinska M., Benes V., Maslinski W. (2020). Global miRNA and mRNA expression profiles identify miRNA-26a-2-3p-dependent repression of IFN signature in systemic sclerosis human monocytes. Eur. J. Immunol..

[14] Mathai S.K., Gulati M., Peng X., Russell T.R., Shaw A.C., Rubinowitz A.N., Murray L.A., Siner J.M., Antin-Ozerkis D.E., Montgomery R.R. (2010). Circulating monocytes from systemic sclerosis patients with interstitial lung disease show an enhanced profibrotic phenotype. Lab. Investig..

[15] Carvalheiro T., Horta S., van Roon J.A.G., Santiago M., Salvador M.J., Trindade H., Radstake T.R.D.J., da Silva J.A.P., Paiva A. (2018). Increased frequencies of circulating CXCL10-, CXCL8- and CCL4-producing monocytes and Siglec-3-expressing myeloid dendritic cells in systemic sclerosis patients. Inflamm. Res..

[16] Carvalheiro T., Lopes A.P., van der Kroef M., Malvar-Fernandez B., Rafael-Vidal C., Hinrichs A.C., Servaas N.H., Bonte-Mineur F., Kok M.R., Beretta L. (2020). Angiopoietin-2 promotes inflammatory activation in monocytes of systemic sclerosis patients. Int. J. Mol. Sci..

[17] Van der Kroef M., Castellucci M., Mokry M., Cossu M., Garonzi M., Bossini-Castillo L.M., Chouri E., Wichers C.G.K., Beretta L., Trombetta E. (2019). Histone modifications underlie monocyte dysregulation in patients with systemic sclerosis, underlining the treatment potential of epigenetic targeting. Ann. Rheum. Dis..

[18] Liu Q., Zaba L., Satpathy A.T., Longmire M., Zhang W., Li K., Granja J., Guo C., Lin J., Li R. (2020). Chromatin accessibility landscapes of skin cells in systemic sclerosis nominate dendritic cells in disease pathogenesis. Nat. Commun..

[19] Hadjicharalambous M.R., Lindsay M.A. (2019). Long Non-Coding RNAs and the Innate Immune Response. Non Coding RNA.

[20] Mariotti B., Servaas N.H., Rossato M., Tamassia N., Cassatella M.A., Cossu M., Beretta L., van der Kroef M., Radstake T.R.D.J., Bazzoni F. (2019). The long non-coding RNA NRIR drives IFN-response in monocytes: Implication for systemic sclerosis. Front. Immunol..

[21] Dolcino M., Tinazzi E., Puccetti A., Lunardi C. (2019). In systemic sclerosis, a unique long non coding RNA regulates genes and pathways involved in the three main features of the disease (vasculopathy, fibrosis and autoimmunity) and in carcinogenesis. J. Clin. Med..

[22] Wang Z., Jinnin M., Nakamura K., Harada M., Kudo H., Nakayama W., Inoue K., Nakashima T., Honda N., Fukushima S. (2016). Long non-coding RNA TSIX is upregulated in scleroderma dermal fibroblasts and controls collagen mRNA stabilization. Exp. Dermatol..

[23] Messemaker T.C., Chadli L., Cai G., Goelela V.S., Boonstra M., Dorjée A.L., Andersen S.N., Mikkers H.M.M., van ’t Hof P., Mei H. (2018). Antisense long non-coding RNAs are deregulated in skin tissue of patients with systemic sclerosis. J. Investig. Dermatol..

[24] Abd-Elmawla M.A., Hassan M., Elsabagh Y.A., Alnaggar A.R.L., Senousy M.A. (2020). Deregulation of long noncoding RNAs ANCR, TINCR, HOTTIP and SPRY4-IT1 in plasma of systemic sclerosis patients: SPRY4-IT1 as a novel biomarker of scleroderma and its subtypes. Cytokine.

[25] Dykes I.M., Emanueli C. (2017). Transcriptional and post-transcriptional gene regulation by long non-coding RNA. Genom. Proteom. Bioinform..

[26] Wang K.C., Chang H.Y. (2011). Molecular mechanisms of long noncoding RNAs. Mol. Cell.

[27] Chen L.-L. (2016). Linking long noncoding RNA localization and function. Trends Biochem. Sci..

[28] Guh C.-Y., Hsieh Y.-H., Chu H.-P. (2020). Functions and properties of nuclear lncRNAs-from systematically mapping the interactomes of lncRNAs. J. Biomed. Sci..

[29] Noh J.H., Kim K.M., McClusky W.G., Abdelmohsen K., Gorospe M. (2018). Cytoplasmic functions of long noncoding RNAs. Wiley Interdiscip. Rev. RNA.

[30] Font-Cunill B., Arnes L., Ferrer J., Sussel L., Beucher A. (2018). Long non-coding RNAs as local regulators of pancreatic islet transcription factor genes. Front. Genet..

[31] Statello L., Guo C.-J., Chen L.-L., Huarte M. (2021). Gene regulation by long non-coding RNAs and its biological functions. Nat. Rev. Mol. Cell Biol..

[32] Frasca L., Lande R. (2020). Toll-like receptors in mediating pathogenesis in systemic sclerosis. Clin. Exp. Immunol..

[33] Ciechomska M., Huigens C.A., Hügle T., Stanly T., Gessner A., Griffiths B., Radstake T.R.D.J., Hambleton S., O’Reilly S., van Laar J.M. (2013). Toll-like receptor-mediated, enhanced production of profibrotic TIMP-1 in monocytes from patients with systemic sclerosis: Role of serum factors. Ann. Rheum. Dis..

[34] Skaug B., Assassi S. (2020). Type I interferon dysregulation in Systemic Sclerosis. Cytokine.

[35] Lafyatis R. (2014). Transforming growth factor β—At the centre of systemic sclerosis. Nat. Rev. Rheumatol..

[36] Cabili M.N., Dunagin M.C., McClanahan P.D., Biaesch A., Padovan-Merhar O., Regev A., Rinn J.L., Raj A. (2015). Localization and abundance analysis of human lncRNAs at single-cell and single-molecule resolution. Genome Biol..

[37] Hu T., Wang F., Han G. (2020). LncRNA PSMB8-AS1 acts as ceRNA of miR-22-3p to regulate DDIT4 expression in glioblastoma. Neurosci. Lett..

[38] Shen G., Mao Y., Su Z., Du J., Yu Y., Xu F. (2020). PSMB8-AS1 activated by ELK1 promotes cell proliferation in glioma via regulating miR-574-5p/RAB10. Biomed. Pharmacother..

[39] Zhang H., Zhu C., He Z., Chen S., Li L., Sun C. (2020). LncRNA PSMB8-AS1 contributes to pancreatic cancer progression via modulating miR-382-3p/STAT1/PD-L1 axis. J. Exp. Clin. Cancer Res..

[40] Harada H., Taniguchi T., Tanaka N. (1998). The role of interferon regulatory factors in the interferon system and cell growth control. Biochimie.

[41] Zhou Y., Lutz P.-E., Wang Y.C., Ragoussis J., Turecki G. (2018). Global long non-coding RNA expression in the rostral anterior cingulate cortex of depressed suicides. Transl. Psychiatry.

[42] Honma K., Tsuzuki S., Nakagawa M., Tagawa H., Nakamura S., Morishima Y., Seto M. (2009). TNFAIP3/A20 functions as a novel tumor suppressor gene in several subtypes of non-Hodgkin lymphomas. Blood.

[43] Lei D., Lv L., Yang L., Wu W., Liu Y., Tu Y., Xu D., Jin Y., Nong X., He L. (2017). Genome-wide analysis of mRNA and long noncoding RNA profiles in chronic actinic dermatitis. BioMed Res. Int..

[44] More S., Zhu Z., Lin K., Huang C., Pushparaj S., Liang Y., Sathiaseelan R., Yang X., Liu L. (2019). Long non-coding RNA PSMB8-AS1 regulates influenza virus replication. RNA Biol..

[45] Shi L., Perin J.C., Leipzig J., Zhang Z., Sullivan K.E. (2011). Genome-wide analysis of interferon regulatory factor I binding in primary human monocytes. Gene.

[46] El Hassan M.A., Huang K., Eswara M.B.K., Xu Z., Yu T., Aubry A., Ni Z., Livne-Bar I., Sangwan M., Ahmad M. (2017). Properties of STAT1 and IRF1 enhancers and the influence of SNPs. BMC Mol. Biol..

[47] Aillaud M., Schulte L.N. (2020). Emerging roles of long noncoding RNAs in the cytoplasmic milieu. Non Coding RNA.

[48] Santini T., Martone J., Ballarino M., Bodega B., Lanzuolo C. (2020). Visualization of nuclear and cytoplasmic long noncoding RNAs at single-cell level by RNA-FISH. Capturing Chromosome Conformation: Methods and Protocols.

[49] Zhang P., Cao L., Zhou R., Yang X., Wu M. (2019). The lncRNA Neat1 promotes activation of inflammasomes in macrophages. Nat. Commun..

[50] Murray R.Z., Stow J.L. (2014). Cytokine secretion in macrophages: SNAREs, rabs, and membrane trafficking. Front. Immunol..

[51] Scala E., Pallotta S., Frezzolini A., Abeni D., Barbieri C., Sampogna F., de Pita O., Puddu P., Paganelli R., Russo G. (2004). Cytokine and chemokine levels in systemic sclerosis: Relationship with cutaneous and internal organ involvement. Clin. Exp. Immunol..

[52] Gourh P., Arnett F.C., Assassi S., Tan F.K., Huang M., Diekman L., Mayes M.D., Reveille J.D., Agarwal S.K. (2009). Plasma cytokine profiles in systemic sclerosis: Associations with autoantibody subsets and clinical manifestations. Arthritis Res. Ther..

[53] Sato S., Hasegawa M., Takehara K. (2001). Serum levels of interleukin-6 and interleukin-10 correlate with total skin thickness score in patients with systemic sclerosis. J. Dermatol. Sci..

[54] Hasegawa M., Fujimoto M., Kikuchi K., Takehara K. (1997). Elevated serum tumor necrosis factor-α levels in patients with systemic sclerosis: Association with pulmonary fibrosis. J. Rheumatol..

[55] De Lauretis A., Sestini P., Pantelidis P., Hoyles R., Hansell D.M., Goh N.S.L., Zappala C.J., Visca D., Maher T.M., Denton C.P. (2013). Serum interleukin 6 is predictive of early functional decline and mortality in interstitial lung disease associated with systemic sclerosis. J. Rheumatol..

[56] Schmidt K., Martinez-Gamboa L., Meier S., Witt C., Meisel C., Hanitsch L.G., Becker M.O., Huscher D., Burmester G.R., Riemekasten G. (2009). Bronchoalveoloar lavage fluid cytokines and chemokines as markers and predictors for the outcome of interstitial lung disease in systemic sclerosis patients. Arthritis Res. Ther..

[57] Crestani B., Seta N., de Bandt M., Soler P., Rolland C., Dehoux M., Boutten A., Dombret M.C., Palazzo E., Kahn M.F. (1994). Interleukin 6 secretion by monocytes and alveolar macrophages in systemic sclerosis with lung involvement. Am. J. Respir. Crit. Care Med..

[58] Trapnell C., Pachter L., Salzberg S.L. (2009). TopHat: Discovering splice junctions with RNA-Seq. Bioinformatics.

[59] Anders S., Pyl P.T., Huber W. (2015). HTSeq—A Python framework to work with high-throughput sequencing data. Bioinformatics.

[60] Love M.I., Huber W., Anders S. (2014). Moderated estimation of fold change and dispersion for RNA-seq data with DESeq2. Genome Biol..

[61] Harrell F.E., Dupont M.C. (2006). The Hmisc Package.

[62] Chen J., Bardes E.E., Aronow B.J., Jegga A.G. (2009). ToppGene Suite for gene list enrichment analysis and candidate gene prioritization. Nucleic Acids Res..

[63] Langfelder P., Horvath S. (2008). WGCNA: An R package for weighted correlation network analysis. BMC Bioinform..

[64] Langfelder P., Luo R., Oldham M.C., Horvath S. (2011). Is my network module preserved and reproducible?. PLoS Comput. Biol..

[65] Schmittgen T.D., Livak K.J. (2008). Analyzing real-time PCR data by the comparative C(T) method. Nat. Protoc..

[66] Wickham H. (2016). ggplot2: Elegant Graphics for Data Analysis.

