# Supplementary material for: Characterization of Long Non-Coding RNAs in Systemic Sclerosis Monocytes: A Potential Role for PSMB8-AS1 in Altered Cytokine Secretion
Source: Int J Mol Sci. 2021 Apr 22;22(9):4365. doi: 10.3390/ijms22094365 (PMC8122435; doi:10.3390/ijms22094365)
Supplement: Supplementary file 1 [file ijms-22-04365-s001.zip › Supplementary Figures.pdf]

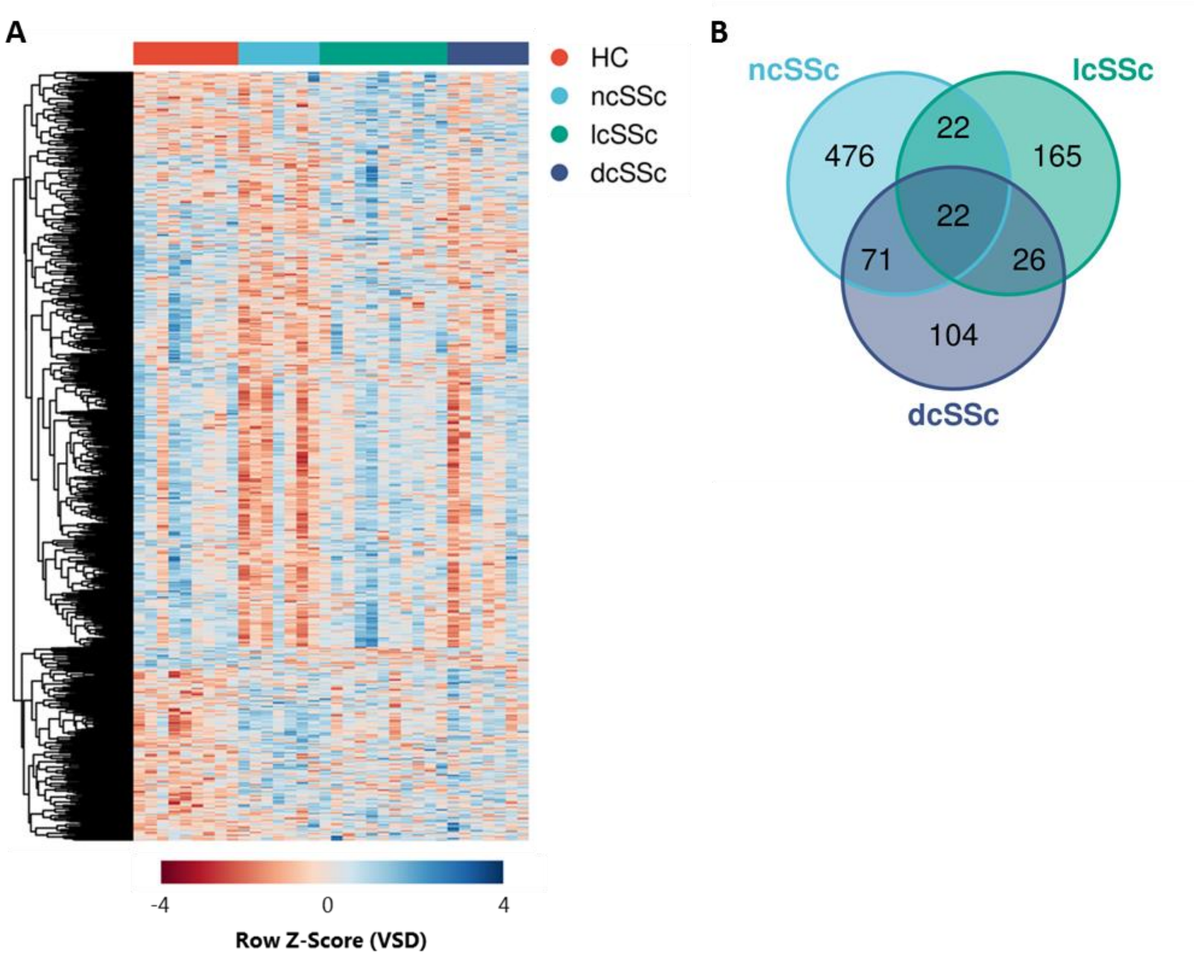

Figure S1. Expression of lncRNAs is altered in SSc monocytes. (**A**) Expression heatmap of lncRNAs significantly modulated in at least one comparison of SSc patients versus healthy controls (log_2(FC) >0.58 or <-0.58, and p-value <0.05). lncRNAs expression is shown as row mean-centered Z-Score of the variance stabilized data (VSD) obtained from RNA-sequencing analysis. (**B**) Venn diagram showing the number of differentially expressed lncRNAs in each SSc patient subset (depicted by different colors) as well as the number of overlapping differential lncRNAs between patient subsets.

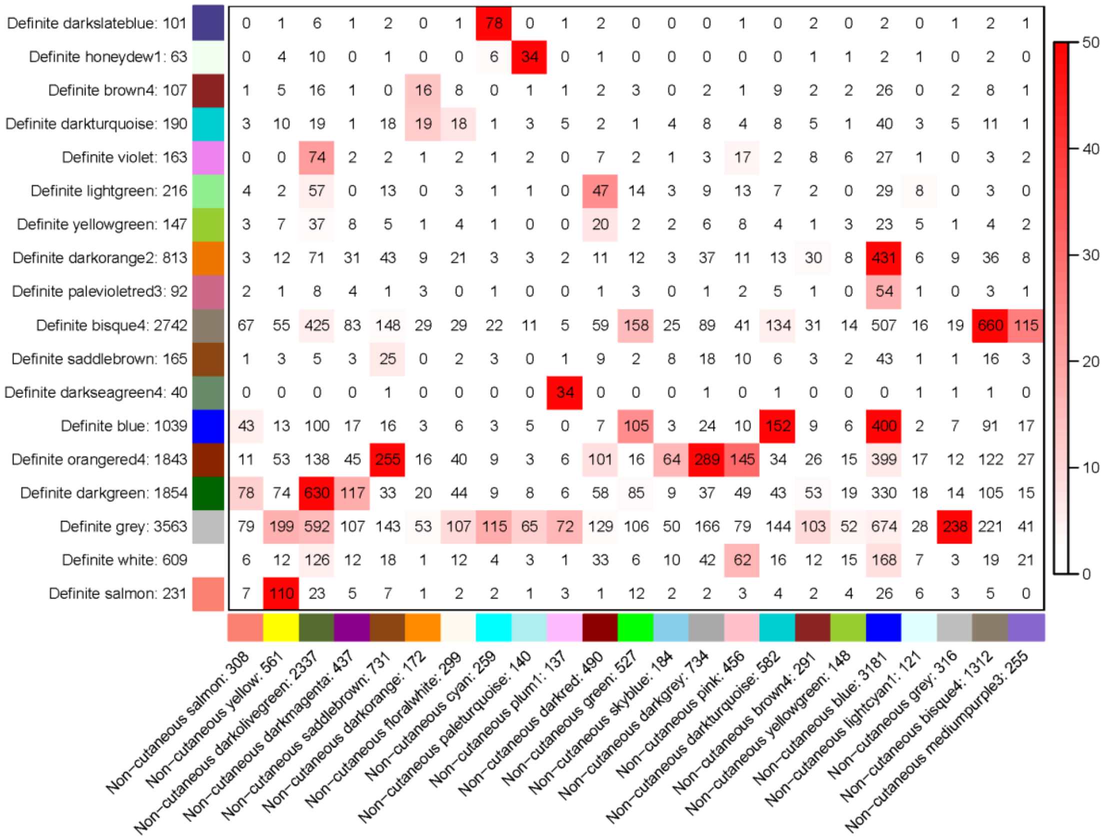

Figure S2. Module overlap between gene co-expression networks identified in the definite and non-cutaneous cohorts. Overlap of all modules from the definite (rows) and non-cutaneous cohorts (columns). Numbers behind modules names indicate the total number of genes in the modules. Numbers in the table indicate the number of genes overlapping between two modules. Coloring indicates the significance of the overlap (Fisher's exact test, $-\log_{10}$(p-value)).

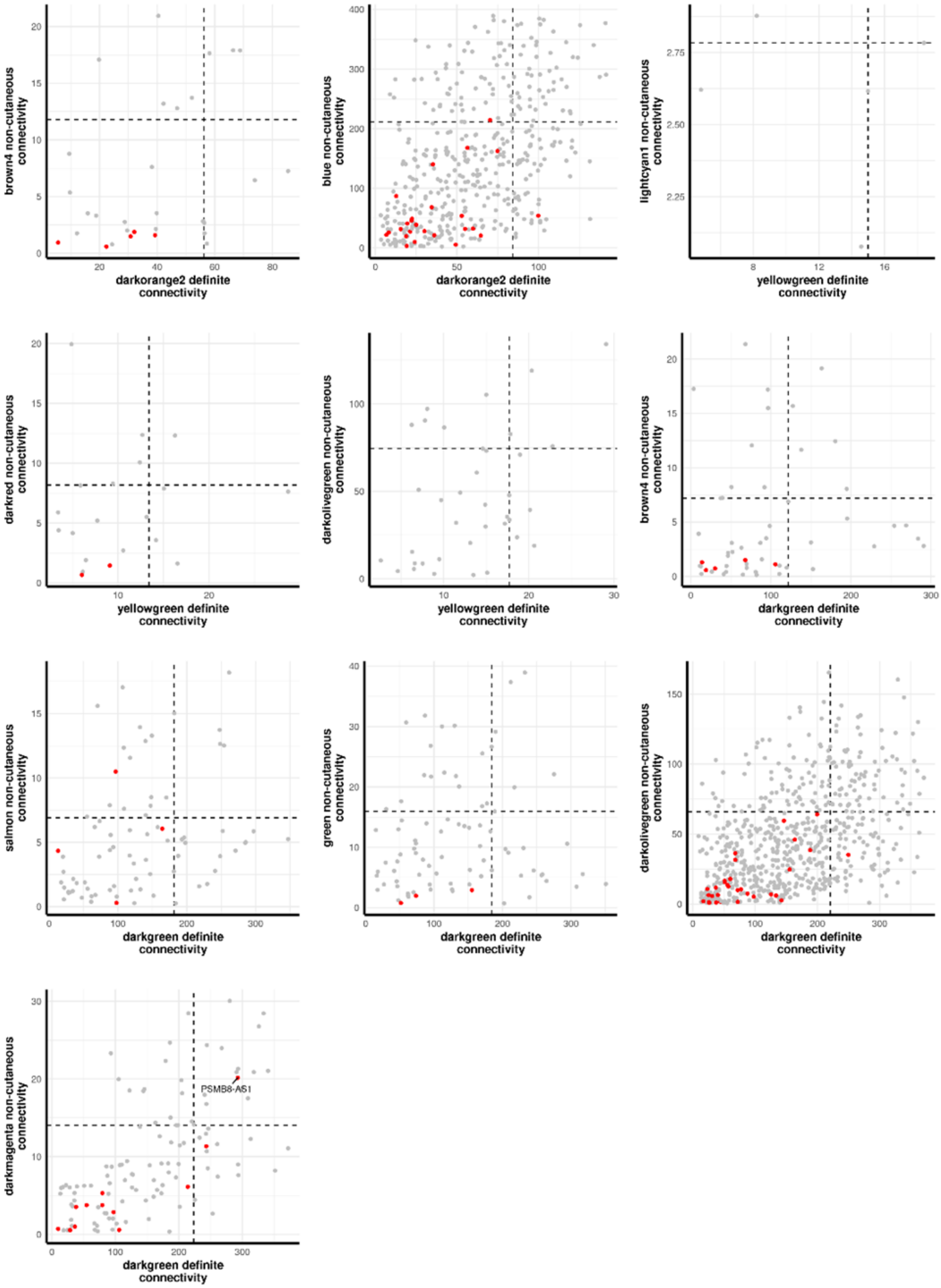

Figure S3. Connectivity overlap of selected reproduced co-expression modules. Intramodular connectivity of genes shared across the selected modules of the definite cohort (x-axis) and corresponding modules of the non-cutaneous cohort (y-axis). Each dot represents one gene, with lncRNAs highlighted in red. The top 25% most connected genes in both the definite and non-cutaneous modules (black dotted lines) were considered as hub-genes.

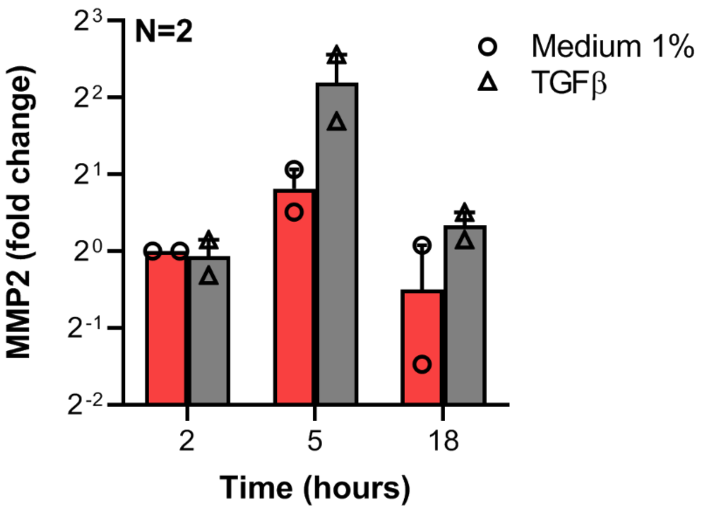

Figure S4. MMP2 expression is induced by TGFβ signaling in monocytes. CD14+ monocytes were cultured for the indicated time points in presence TGFβ (grey bars), or left untreated (medium control, red bars). PSMB8-AS1 expression was analyzed and expressed as fold change over the medium control at 2h. Data are shown as mean+/-SEM.

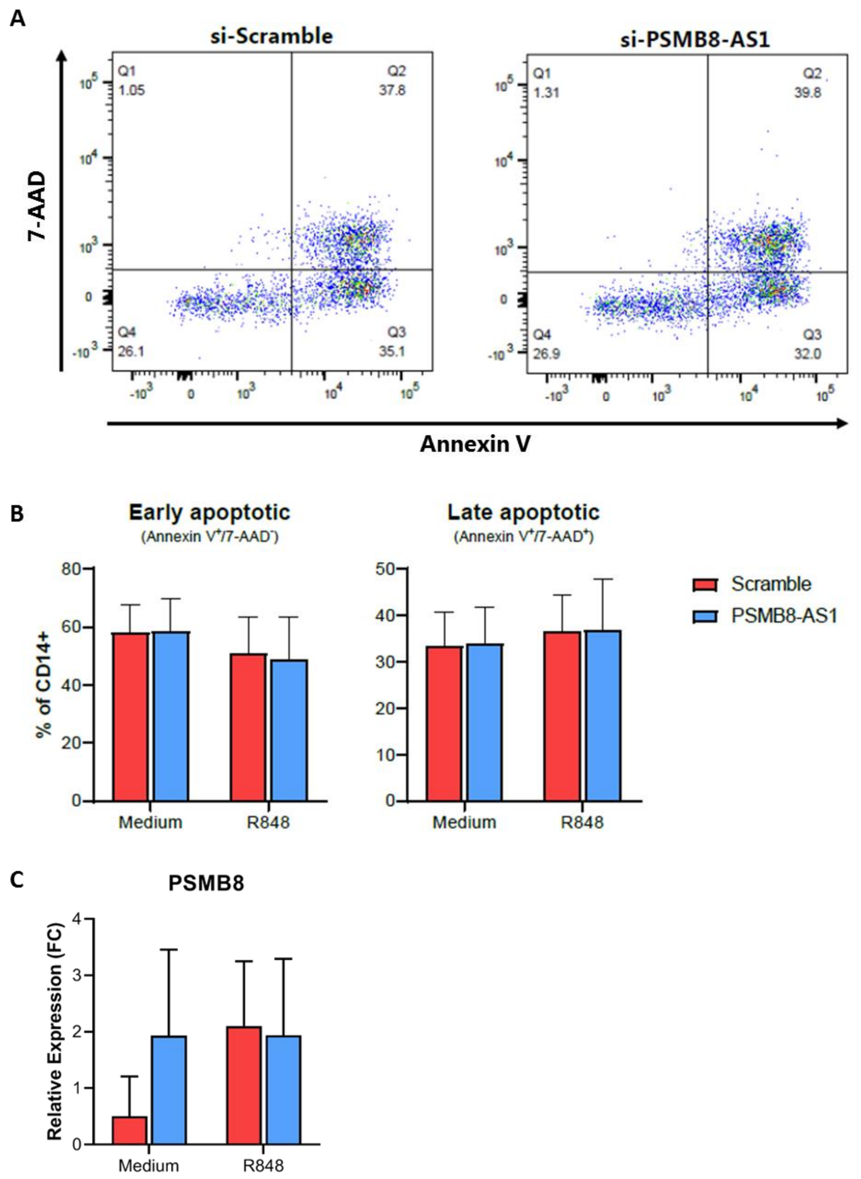

Figure S5. Silencing of PSMB8-AS1 does not affect apoptosis or PSMB8 expression in healthy monocytes. (**A**) Representative FACS plots of viability analysis of monocytes transfected with si-PSMB8-AS1 (right panel) or scramble siRNA (left panel). The percentage of viable (Q4), early apoptotic (Q3) and late apoptotic (Q2) cells, based on Annexin V (x-axis) and 7-AAD (y-axis) staining is indicated in the corresponding quadrants. (**B**) Bar graphs showing the percentage of early apoptotic (left panel) and late apoptotic (right panel) within the fraction of CD14+ monocytes. Cells were transfected with si-PSMB8-AS1 (blue) or scramble siRNA (red) , and either left unstimulated or treated with R848 (N=5). (**C**) Expression of PSMB8 was analyzed by RT-qPCR and expressed as relative expression (y-axis, FC=fold change) compared to medium scramble control (N=3).
